# Innovative Integration of Arrayan (*Luma apiculata*) Extracts in Chitosan Coating for Fresh Strawberry Preservation

**DOI:** 10.3390/ijms241914681

**Published:** 2023-09-28

**Authors:** Sergio Benavides, Wendy Franco

**Affiliations:** 1 School of Nutrition and Dietetics, Faculty of Health Care Sciences, Universidad San Sebastián, Concepción 4080871, Chile; 2 Agro-Food and Applied Nutrition Research Center, Adventist University of Chile, Chillan 3780000, Chile; 3 Chemical Engineering and Bioprocess Department, Pontificia Universidad Católica de Chile, Av. Vicuña Mackenna 4860, Macul, Santiago 7820436, Chile; 4 Departamento de Ciencias de la Salud, Carrera de Nutrición y Dietética, Pontificia Universidad Católica de Chile, Av. Vicuña Mackenna 4860, Macul, Santiago 7820436, Chile

**Keywords:** edible coating, chitosan, Arrayan, strawberries, shelf-life

## Abstract

Strawberries are a rich source of vitamins and antioxidants, among other nutrients, but they are highly susceptible to mechanical injuries, dehydration, and microbial spoilage, and thus have a limited post-harvest shelf-life. Bioactive edible coatings have been studied to decrease or prevent these damages. In this study, ethanolic extracts of Arrayan (*Luma apiculata*), a traditional berry from the south of Chile, were used to enrich a chitosan-based edible film and coat fresh strawberries. A long-term storage (10 °C) study was conducted to determine the strawberries’ weight loss, microbial stability, fruit firmness impact, and antioxidant activity. Later, a sensory panel was conducted to determine overall consumer acceptance. Our results show that the bioactive coating inhibited the growth of different pathogenic bacteria and spoilage yeast. In the stored strawberries, the weight loss was significantly lower when the bioactive coating was applied, and the samples’ firmness did not change significantly over time. Microbial growth in the treated strawberries was also lower than in the control ones. As expected, the antioxidant activity in the coated strawberries was higher because of the Arrayan extract, which has high antioxidant activity. Regarding sensory qualities, the covered strawberries did not show significant differences from the uncoated samples, with an overall acceptance of 7.64 on a 9-point scale. To our knowledge, this is the first time an edible coating enriched with Arrayan extracts has been reported as able to prevent strawberries’ decay and spoilage.

## 1. Introduction

Fruits constitute an important part of the human diet. They are a source of several minerals, vitamins, sugars, and fiber, among other biomolecules. Huang et al., 2012, reported that strawberries, for instance, are a rich source of antioxidants, including vitamin C, folate, and polyphenolic compounds [1]. However, the fruit is very perishable since it is susceptible to microbial decay, dehydration, and mechanical injury [2]. Strawberries are particularly sensitive to storage, with the mean shelf-life of the fruit 3 to 10 days at 10 °C.

An alternative to alleviate this problem is using bioactive coatings made with natural compounds. Bioactive coatings can be elaborated with different edible biopolymers and can incorporate plant extracts with antimicrobial and/or antioxidant properties. These coatings can inhibit microbial growth, inactivate spoilage enzymes, and limit dehydration [3,4,5]. A common biopolymer used in the production of food coatings is chitosan [6,7]. This biopolymer has been extensively used for the decay protection of several fresh food products [8], and it has also been reported as an effective barrier in strawberries [9,10,11,12]. Chitosan is a polysaccharide formed by β(1-4)-d-glucosamine and *N*-acetyl-d-glucosamine, which is obtained from mushrooms or the shells of crustaceous [13]. The compound is non-toxic (Generally Recognized as Safe, GRAS), biodegradable, and shows antimicrobial properties [6]. In several studies, chitosan coatings are enriched with bioactive natural molecules (essential oils, botanical extracts, and organic acids, among others) to enhance their bioactive properties [14,15,16].

Different bioactive films composed of chitosan and other molecules have been reported as able to control or prevent decay in fruits. A blend of chitosan and pullulan in equal parts (50:50), combined with an extract from pomegranate peel (at a concentration of 0.02 g/mL), was utilized as an edible coating in mango fruits. This coating proved effective in minimizing the loss of weight and preserving various quality attributes of the mango fruits over a span of 18 days during postharvest storage at a temperature of 4 °C. The characteristics that were maintained include total soluble solids (TSS), pH, firmness, phenolic content, and the antioxidant activity of the fruits. [17].

In another study, a coating composed of chitosan (at a concentration of 1.5% by weight) infused with an extract from hairy fig (*Ficus hirta Vahl.*) was used to coat “Newhall” navel oranges. This treatment exhibited the lowest decay rate (5.2%), minimized weight loss (5.16%), and reduced the malondialdehyde content. Furthermore, it bolstered the function of protective enzymes like superoxide dismutase, peroxidase, chitinase, and -1,3-glucanase throughout a span of 120 days in cold storage [18]. Furthermore, when guavas (Allahabadsafeda variety) were coated with a mixture of chitosan (at a concentration of 1% by weight) and alginate (at a concentration of 2% by weight), along with pomegranate peel extract (at a concentration of 1% by weight), noticeable reductions were observed in the losses of ascorbic acid (29%), total phenolics (8%), total flavonoids (12%), and antioxidant activity as measured through DPPH (12%) and FRAP (9%) assays. These effects were sustained over a period of 20 days while the coated guavas were stored at a temperature of 10 °C [18].

Bioactive chitosan coatings have been also used to prevent strawberry decay, especially when the coating is enriched with high-antioxidant plant extracts.

**Figure 1:**
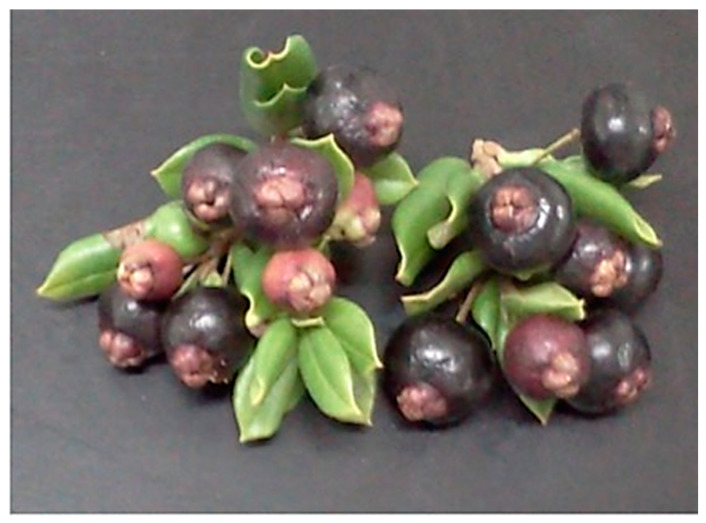
Arrayan fruits.

Plant extracts, especially those having a high antioxidant composition, such as berries, are an interesting alternative as a food additive for preserving fresh food products [17]. Different wild berries are grown in the south of Chile. Among them, Arrayan (*Luma apiculata*) is an endemic fruit with interesting properties to be considered as a natural food additive. Arrayan belongs to the *Myrtaceae* family and is an endemic tree cultivated in Chile from the Valparaíso to the Aisén Regions (latitude 33° to 45°). The tree has red-brown stems, with regular leaves, and black to purple edible fruits with a mild and pleasant taste [18,19] (Figure 1). The fruit is commonly used for infusions, meal preparations, and ointments [20].

Little information is available in relation to Arrayan extract properties and/or applications. The fruits have been described as a rich source of phenolic compounds with high antioxidant activity [19,21,22,23]. Polyphenols are a group of naturally occurring compounds found in plants, known for their antioxidant and antimicrobial properties and potential health-promoting effects. They are responsible for the vibrant colors of many fruits, vegetables, and herbs. Anthocyanins are the most abundant polyphenols in Arrayan extracts. Flavanols have been also reported, but in lower concentrations [18,20,24]. Arrayan polyphenols have been reported as able to inhibit pathogenic bacteria such as *Pseudomonas* sp. and *Staphylococcus* sp. [18,19].

Despite these interesting properties, to our knowledge, little information has been reported regarding potential food applications of Arrayan in terms of the decay protection of fruits. Therefore, this work aimed to design a bioactive coating based on a chitosan solution enriched with Arrayan fruit ethanolic extract to improve the shelf-life of fresh strawberries during cold storage.

## 2. Results and Discussion

### 2.1. Phenolic Composition of Arrayan Extracts

Polyphenols make up the majority of secondary compounds found in berries. The most abundant and varied categories of phenolic substances discovered in berries include anthocyanins, followed by flavones and flavonoids [25]. Six types of anthocyanins have been identified in berries, including cyanidin, delphinidin, malvidin, peonidin, pelargonidin, and petunidin [20]. For Arrayan, in specific, delphinidin, petunidin, peonidin, and malvidin have been reported before [18,22,23]. In our study, Peonidin-3-galactoside was the major anthocyanin present (438 ± 7.00 mg/L) in the Arrayan extracts, followed by Petunidin-3-arabinoside (121 ± 3.00 mg/mL), while Peonidin-3-arabinoside and Malvidin-3-arabinoside were encounter found in concentrations lower than 100 mg/L (Table 1). As for the flavonoids, quercetin has been also reported for in Arrayan extracts [20,24], however, in lower concentration than the one reported here (718 mg/L mg/L).

These molecules have been previously reported as having functional bioactivities that include the prevention or inhibition of microorganisms. Quercetin has been reported as a broad antimicrobial agent capable of inhibiting both bacteria and fungi [26]. Anthocyanins are a class of natural pigments found abundantly in various berries. These water-soluble compounds are responsible for the vibrant red, purple, and blue colors exhibited by these fruits. Beyond their role in coloration, anthocyanins have garnered significant interest in scientific research due to their potential health benefits, particularly their antibacterial activity [27,28].

### 2.2. Antimicrobial Activity

Foodborne bacteria represent a constant risk for human consumption. The presence of coliforms such as *Salmonella* sp. and *Escherichia coli* has been associated with ready-to-eat fruits [29]. In 2011 and 2016, outbreaks associated with *E. coli* O157:H7 in strawberries were reported [30]. Additionally, there have been connections to *Salmonella* sp. outbreaks, as well [31]. On the other hand, *Staphylococcus aureus* and *Listeria* sp. contaminations are associated with poor manufacturing practices of ready-to-eat fruits [32].

Microbial strawberry decay is also associated with fungi growth, in which yeasts play an important spoilage role [33]. Therefore, the antimicrobial capability of free extracts and the BC coating were tested against selected foodborne bacteria and yeasts.

The antimicrobial activity of the free extract (FE), a 1% *w*/*v* chitosan solution (CH), and the bioactive coating (1% *w*/*v* chitosan + 0.8% *w*/*v* free extract, BC) were determined in vitro. All the treatments inhibited the growth of bacteria and yeasts (Table 2). As expected, the FE showed, in general, significantly greater antimicrobial effects than the CH or BC. The antimicrobial activity was transversal to either Gram-positive (*St. aureus* and *L. inoccua*) or Gram-negative (*S. typhimurium* and *E. coli*) bacteria, as well as yeasts (*P. kluyverii* and *M. pulcherrima*).

Viktorová et al. (2020) studied the antimicrobial effects of a methanolic Arrayan extract over *Pseudomonas aeruginosa* and *St. aureus*, establishing for the latter an IC50 (median inhibitory concentration) between 0.354 and 0.385 mg/mL [20]. On the other hand, Treulen et al. (2019) established the MIC of an Arrayan extract for *St. aureus* between 1 and 10 mg/mL, consistent with our study, where a dose used for BC of 0.8% *w*/*v* (8 mg/mL) was established [34]. In a similar manner, Araya-Contreras et al. (2019) determined that hexane Arrayan extracts showed antibacterial activity against *St. aureus*, *St. epidermidis*, *I. saprophyticus*, *Enterococcus* sp., *A. baumanii*, *Pseudomona aeruginosa*, and *E. coli* at concentrations greater than 100 μg/mL [19].

The presence of bioactive compounds, such as flavonoids, tannins, and essential oils, in the extract is thought to contribute to this antibacterial effect [18]. These compounds have demonstrated the ability to disrupt bacterial cell membranes, inhibit enzymatic activities crucial for bacterial survival, and interfere with bacterial quorum sensing, leading to reduced bacterial growth and virulence [35].

The BC’s antimicrobial activity was significantly lower than the FE but higher than the CH alone. This can be explained by the slowing effect generated by the chitosan matrix on the bioactive agent (FE) release. This effect has already been identified in other studies, where a polymeric matrix affects the release rate of an antimicrobial or antioxidant agent, decreasing its initial impact, but maintaining a certain persistence over time [36,37,38,39,40]. This can be advantageous in terms of ensuring a regulated activity over time, especially during food storage. However, a minimum release threshold must be established to generate the expected bioactive effect.

### 2.3. Physical and Chemical Evolution during Cold Storage

The effect of the BC on fresh strawberries’ shelf life was assessed by coating fresh strawberries with the FE (0.8% *w*/*v*), the CH (1.0% *w*/*v* chitosan), or the BC (1.0% *w*/*v* chitosan + 0.8% *w*/*v* FE), and then storing at 10 °C for 21 days. During this time, different parameters were determined every 7 days.

#### 2.3.1. Weight Loss

**Figure 2:**
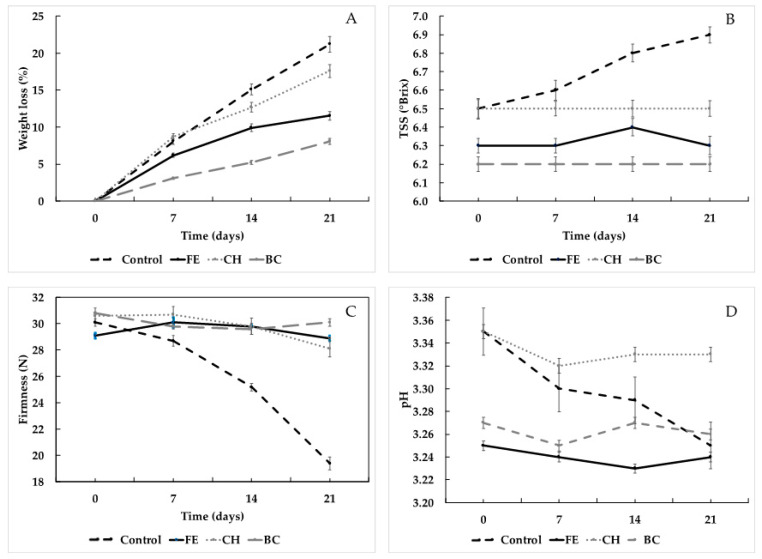
Physical and chemical changes in the treated strawberries (FE, CH, and BC) and control (without treatment) during storage (21 days at 10 °C). (**A**) Weight loss; (**B**) total soluble solids; (**C**) firmness; (**D**) pH.

During storage, water loss, and therefore weight loss, is a very common problem that impairs the quality of the strawberries. Figure 2A shows the weight loss experienced by the experimental strawberries for 21 days (3 weeks). Overall, the water loss in all the treated samples increased with time. However, the samples treated with the bioactive coating showed a significantly lower weight loss percentage than the other treatment and the control samples. The greatest weight loss occurred for the control samples (without treatment), reaching a loss of 8% in the first week, close to 15% in the second, and over 20% in the third, while the strawberries coated either with the CH or the BC showed lower weight loss trends. The lowest weight loss was observed when the strawberries were coated with the BC, where, after 3 weeks of storage, weight loss was close to 7%, with no significant differences being observed with the control group at week 0, suggesting that the addition of the FE in a polymeric matrix significantly increases the stability of fresh strawberries.

Muley and Singhal (2020) demonstrated that by using chitosan coatings, it is possible to control weight loss in strawberries [10]. In a test carried out at 5 °C, they managed to reduce the weight loss by approximately 50% compared to the control group on day 5 of storage. By day 7, the coated strawberries had only lost 13% of their initial weight, a higher loss than our results, in which, on day 7, the samples with BC had only lost 7% in weight at 10 °C [10], while Petriccione et al. reported a 7–9% weigh loss when different strawberries were coated with chitosan [41] after 10 days of storage. The bioactive coating used in our study was enriched with a rich polyphenolic extract that can also aid in the retention of water in the fruit, and therefore contribute to the control of weight loss. Riaz et al. (2020) suggested that slower weight losses can be attributed to a decrease in the hydrophilicity nature of CH, due to the incorporation of polyphenol-rich extracts [42].

#### 2.3.2. Firmness

Figure 2C shows the results observed regarding firmness during cold storage. The control samples showed higher firmness loss, that is, the strawberries without treatment. On the other hand, in the treated samples, little difference was observed, showing values between 28 to 30 N of rupture force. There is a close relationship between the water content in fruit and its resistance to rupture. A higher water content increases turgor. In the opposite direction, a lower water content implies less turgor and, consequently, less firmness [43].

The samples treated with the BC showed lower weight loss values, which allowed them to maintain greater firmness. In percentage terms, the control group lost 35.5% firmness, while the BC treatment only lost 2.3%. Saleh and Abu-Dieyeh (2022) studied the effects of a chitosan film containing *Prosopis juliflora* extract to increase the shelf-life of strawberries [14]. They concluded that their control group (strawberries without treatment) showed up to 30.2% firmness loss, whereas the strawberries treated with the coating had no difference in terms of firmness between days 0 and 21. These results are consistent with those reported in this study. Riaz et al. (2020) reported that when strawberries are coated with a polyphenol-rich chitosan coating, the fruits retain firmness after six days of cold storage. This can be possible because the coating decreases the respiration rate, and therefore decreases the fruit ripening and senescence [44].

#### 2.3.3. Total Soluble Solids (TSS)

Figure 2B shows the variations in total soluble solids during the storage period. The results indicate that the control group significantly increased the concentration of TSS, while no significant variations were observed for the FE, CH, or BC treatments. The variation in the control group might also be related to the weight loss of the strawberries [Figure 2A] since, during storage, the strawberries lose water by exudation, and sugars and other solids are concentrated, resulting in increases in °Brix. In a similar study by Saleh and Abu-Dieyeh (2022), the same phenomenon was evidenced between control groups of strawberries (without treatment) and strawberries treated with an extract of *Prosopis juliflora* and chitosan [14]. They demonstrated that the control group increased TSS by 8.9%, while the coated group only increased by 1.5%. They also associated the increase in TSS with the weight loss of the strawberries during storage.

Changes in soluble solids can also be attributed to the respiratory rates of the stored strawberries. Ayala-Zavala et al. (2004) reported that, in storage at 10 °C, a higher loss in TSS was observed, compared to storage at 0 and 5 °C. The authors attributed that, at 10 °C, the respiratory rate of the fruits was increased, explaining in part the changes in TSS [45]. When strawberries are treated with bioactive coatings, the respiratory rate of the fruit decreases. Esghi et al. (2014) reported slower respiration when strawberries were coated with a nano-chitosan suspension [46]. Garcia et al. (2010, 2011) used a cassava starch edible coating and verified a decrease in the respiration rate and an increase in water vapor resistance, as well as reduced weight loss and mechanical property loss [47,48].

#### 2.3.4. pH

During the cold storage, the samples treated with the FE, the CH solution, and the BC showed little variation in pH, maintaining values close to the initial values (3.25, 3.75, and 3.67, for the FE, the CH solution, and the BC, respectively), while, in the control samples, decreases in pH were observed (Figure 2D). Changes in pH during the storage of fresh fruits are commonly associated with the spoilage process, in which bacteria produce organic acids due to bacterial proliferation [49]. This result correlates with the microbial quality observed during the storage, in which the control samples showed greater microbial loads (Table 2).

### 2.4. Microbial Stability during Strawberries Cold Storage

The shelf-life of a food is determined in part by its microbiological stability, that is, the ability to limit or prevent deteriorating microbial proliferation. In that sense, the BC and its components (FE and CH) were evaluated regarding their ability to restrict the growth of aerobic mesophilic microorganisms and fungi (molds and yeasts). The effect was measured after 3 weeks of cold storage, and the results are shown in Table 3.

It can be seen that both aerobic mesophilic microorganisms and fungi were able to increase freely in the control group, reaching 4.32 log CFU/g at the end of the storage, well above the concentrations observed in the treated strawberries. The application of BC was significantly more effective than the rest of the treatments since, in the third week, the microbial population did not exceed the concentration of 1.7 log CFU/g, with no significant differences from the control group on day 0. Similar results were observed for yeast growth, in which the BC-coated samples showed 1.05 log CFU/g after 3 weeks of storage, with no significant differences from the concentrations at the beginning of the experimentation while, in the control sample, the final yeast concentrations reached values higher than 4 log CFU/g (Table 3). The use of BC has been reported as an efficient way to prevent microbial spoilage in different food products since the bioactive compound embedded in the polymer allows for a controlled release of the antimicrobial, which prevents microbial proliferation [49,50]. Berry extracts, and especially their anthocyanins, have been reported as an interesting alternative for the development of bioactive films, which enable the control of microorganisms (both fungi and bacteria) to maintain freshness and extend food shelf-life [51]. The Arrayan extract studied here is rich in these molecules, therefore making it a desirable additive for the development of bioactive coatings.

### 2.5. DPPH Radical Scavenging Assay and ORAC Antioxidant Activity

Berries are commonly a good source of antioxidants responsible for the oxidation of free radicals, acting as oxygen scavengers. As the fruit ripens, the antioxidant activity increases; however, this activity is lost as the fruit decays. In our study, we propose to use a BC enriched with Arrayan extract, which is rich in polyphenols, anthocyanin, and flavonoids [24], as a means to protect against antioxidant loss in the strawberries.

Two techniques were used to determine the oxygen-scavenging activity of the antioxidants DPPH and ORAC. We measured these two in the stored strawberries at the beginning and end of the storage (Table 4).

In the case of the control group, the ability to control DPPH decreased from 167.2 to 49.50 μmol of TE·100 g/dw, that is, a 70% decrease in the antioxidant activity of strawberries. On the other hand, for the group with the BC treatment, the ability to control DPPH only decreased by 16% (from 315.4 to 264.1 μmol of TE·100 g/dw). Something similar occurred in the ORAC measurement, where the control group had a fall of 18% in Trolox equivalent terms, against only 5% of the group of strawberries treated with BC. On the other hand, it was possible to show that the antioxidant capacity of the control reached a Trolox equivalent of 61.1 at the end of 3 weeks, compared to the treated group of 289 at the same time. These results are consistent with those reported by Muley and Singhal (2020), who showed a 27% drop in the control capacity of DPPH in 8 days of cold storage [10]. It should be noted that they used a coating based on chitosan and wheat protein, without adding any bioactive, which would indicate that the barrier effect of the biopolymers can protect and retain the antioxidants present in the fruit.

Our results differ from those reported by Popescu et al. (2022) [12] and Saleh and Abu (2022) [14], which reported increases in the antioxidant activity of strawberries coated with bioactive films. The increases were associated with fruit maturity [12,14]. In our study, the storage was carried out at 10 °C, which could have prevented the fruit ripening.

### 2.6. Total Phenolic Content (TPC), Ascorbic Acid, Flavonoids, and Anthocyanins

Antioxidant activity is usually related to the presence of molecules such as polyphenols, anthocyanins, flavonoids, and ascorbic acid, compounds in which strawberries are very rich. Protecting these functional molecules during storage is an important factor in maintaining the nutritional characteristics of the strawberries. In that sense, the ability of BC to prevent the loss of antioxidant agents was evaluated. Table 5 shows the variation in flavonoids, polyphenols, anthocyanins, and ascorbic acid in strawberries coated with the BC after 3 weeks of cold storage. A drop in concentration was observed over time for all the molecules, which is to be expected given that they progressively oxidize due to contact with air. However, there are significant differences in losses between the control group (without treatment) and strawberries treated with BC.

Regarding the flavonoids, it can be seen that the control group showed a loss of up to 49% after 21 days of storage, while in the BC-treated strawberries, it was only 14%. Regarding the polyphenols, the drop in the control group was greater, losing up to 72% of these antioxidant agents, compared to the treated strawberries, which lost only 18%. Something similar occurred with the ascorbic acid, where the control group lost 45% of the compounds, compared to the treated strawberries, with losses of only 17%. Finally, the anthocyanins show losses of 16% for the control group and less than 1% for the treated strawberries. This might be due to the protective barrier of the BC (chitosan + Arrayan extract) against oxidative reactions, which reduces the loss of the antioxidant compounds. Different studies have shown similar behavior when using films or coatings with the incorporation of antioxidant agents for the preservation of food [10,52,53,54].

### 2.7. Sensory Analysis

**Figure 3:**
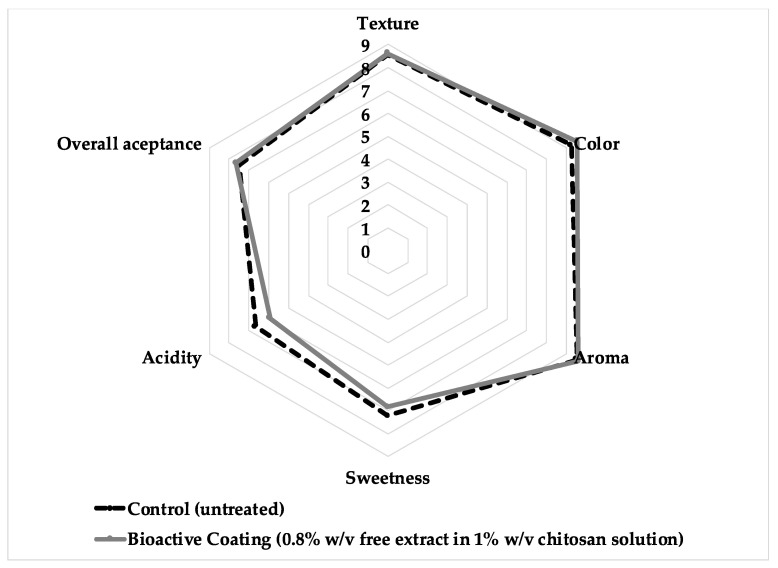
Sensory appreciation of strawberries coated with the bioactive coating (0.8% *w*/*v* free Arrayan extract in 1% *w*/*v* chitosan solution). Attributes were evaluated on a 9-point hedonic scale, where 1 was “dislike extremely”, and 9 was “like extremely”.

Twenty-five panelists analyzed strawberries freshly coated with the BC to determine the effect on the treated samples’ texture, color, aroma, sweetness, and acidity. The results showed no significant differences in any of the characteristics between the untreated and the treated samples (Figure 3), indicating that the application of the bioactive coating does not change the natural characteristics of the fresh strawberries. Furthermore, the overall acceptance of the strawberries was close to 8 for both samples.

## 3. Materials and Methods

### 3.1. Plant Material

Arrayan branches with leaves and ripe fruits were collected from the Valparaiso Region (33°02′18.58″ S; 71°29′48.04″ W) and transported to the Microbial Food Laboratory (Pontifical Catholic University of Chile) in coolers provided with dry ice packs. The leaves and fruits were manually separated at the laboratory, washed with a sodium hypochlorite solution (100 mg/L) (J.T. Baker, Mexico D.F., Mexico), and washed twice with distilled water.

### 3.2. Extracts and Coatings Preparation

The extracts were prepared as Viktorová et al. (2020) described, with a few modifications. Briefly, the sanitized fruits were oven-dried (Thermo Scientific PR205045G) at 60 ± 5 °C for 48 h, finely ground, and pulverized using a food processor to achieve a particle size of 0.3–0.5 mm [18]. The powder was mixed with 100% ethanol (J.T. Baker, Mexico). The mixture was shaken (Slow-Speed Orbital Shaker, Cole-Palmer, Vernon Hills, IL, USA) at 100 rpm at room temperature for 24 h. Then, the extraction mixture was filtered using a Whatman No. 1 filter (Whatman International, Maidstone, UK). The filtered extracts were dried under reduced pressure at 40 °C using a rotary evaporator (Büchi, Labortechnik AG, Flawil, Switzerland). The crude extracts were filter-sterilized (0.45 μm, Nalgene, Millipore, Milwaukee, WI, USA) and stored at −20 °C until experimentation. For the experiment, three coating solutions were analyzed: (i) free extract solution (FE: 0.8% *w*/*v*), (ii) chitosan solution (CH: 1.0% *w*/*v*), and (iii) bioactive coating (BC: 0.8% *w*/*v* free extract in 1.0% *w*/*v* chitosan solution). Based on preliminary studies, the selected concentration showed the minimal concentration for bacteria and fungi control.

#### Free Extract Polyphenolic Composition

The quantification of specific polyphenols was carried out following the protocol reported by Allcca-Alca (2019). Briefly, using Millipore filter type GV (0.22 µm), twenty µL of the filtered extract (0.22 µm, Millipore filter) was analyzed in an HPLC system (Waters 2695, Waters, Milford, MA, USA) equipped with a photodiode array detector (PAD) (Waters, Milford, MA, USA). An X-terra RP_18_ (5 µm, 250 mm × 4.6 mm) column (Waters, Milford, MA, USA) was used for phenolic separation at 30 °C. The mobile phase was made from water:formic acid (95:5, *v*/*v*, pH 2) and acetonitrile, used in a gradient. A flow rate of 0.5 mL/min was used. Polyphenols were identified and quantified by comparing the retention time and area under the curve of different standards. The analyses were performed in triplicate, and the results were expressed as mg of specific polyphenol per gram of dried sample [55].

The anthocyanin identification was conducted following the protocol reported by Fuentes et al. (2016) in methanol and methanol–HCl extract of Arrayan berries with an elution gradient of water/glacial acetic acid/phosphoric acid [56]. Detection wavelengths were 520 and 280 nm, respectively. The flow rate was 1 mL/min, column temperature was 40C, and injection volume was 20 mL. The quantification was done by comparison with standards [56].

### 3.3. Coatings’ In-Vitro Antimicrobial Activity

Four food-born bacteria: *Staphylococcus aureus* (ATCC 6538), *Listeria innocua* (ATCC 33090, *Escherichia coli* (ATCC 25922), and *Salmonella typhimurium* (ATCC 14028), and two deteriorative yeasts (*Pichia kluyverii* and *Metchsnikowia pulcherrima*) were used for the antimicrobial susceptibility test. The bacteria were obtained from the American Type Culture Collection (ATCC); while the spoilage yeasts were isolated from spoiled strawberries and stored in the culture collection at the Microbiology and Food Fermentation Laboratory at Pontificia Universidad Católica de Chile, (Santiago, Chile).

The disk diffusion method was followed [57]. Briefly, the microorganisms were overnight cultured in Mueller Hinton Broth (MHB, Oxoid, Thermofisher, Hampshire, England) to achieve a cell concentration of about 6 log CFU/mL. Then, 500 μL of the active culture was spread onto Mueller Hinton Agar (MHA, Oxoid, Thermofisher, Hampshire, England). Blank discs (BBL, Dilaco, Santiago, Chile.) were impregnated with 20 μL of each coating solution and placed in the inoculated agar plates. A blank disc (negative control), penicillin, and chloramphenicol discs were used as positive controls for the bacterial experiments, and a tioconazole disc was used for the yeast cultures (Oxoid, Thermofisher, Hampshire, UK). Inhibition halos were measured after a one-day incubation at 37 °C for the bacterial experiment and two days at 25 °C for the yeast ones.

### 3.4. Shelf-Life Study (Physical, Chemical, and Microbiological Changes)

Fresh strawberries were purchased at “La Vega” Farmer´s Market (Santiago, Chile). The strawberries were screened for decay and/or bruises upon arrival at the laboratory. Those that showed visual defects were discarded. The experimental design reported by Saleh et al. (2022) was followed [14]. Briefly, the decay-free strawberries were divided into four batches of 30 individual berries.

- Control: Untreated strawberries.
- Free extract (FE): Strawberries sprayed with free extract solution 0.8% *w*/*v*.
- Chitosan solution (CH): Strawberries dipped in 1.0% *w*/*v* chitosan solution.
- Bioactive coating (BC): Strawberries dipped in the bioactive coating (1.0% *w*/*v* chitosan + 0.8% *w*/*v* free extract).

A randomized design was used for the shelf-life study in which five samples made one experiment unit (one replicate), and each experimental condition had six replicates. The coated strawberries were left to air-dry over a sterilized rack at room temperature. Later, each replicate was placed in a sanitized plastic clamp container. The samples were stored for 21 days at 10 ± 2 °C and an ambient humidity of 60 ± 5% (Hygrometer with datalogging, Fisherbrand, Waltham, MA, USA) and analyzed at the beginning of the experiment and, after that, every 7 days. Weight loss, firmness, total solids concentration, pH, and microbial proliferation were measured to determine the impact on shelf-life.

#### 3.4.1. Weight Loss

The strawberry samples were weighed at the beginning of each experiment (day 0), and then the weight was measured at the time of sample collection up to 21 days of storage. The difference between the initial and final weights determined the weight loss. An average percent loss was calculated [58].

#### 3.4.2. Firmness

The firmness of the treated strawberries was evaluated in terms of the force required to rupture the rind of the fruits. A texture analyzer (TA-XT2, TA Instruments, Crawley, UK) was used for this. The measurement was carried out in the central upper quadrant area of the strawberries using a cylindrical probe (aluminum, diameter: 5 mm). The test speed was fixed at 600 mm/min [59].

#### 3.4.3. pH and Titratable Acidity (TTA)

The pH was measured with a pH meter (Mettler Toledo, Columbus, ON, USA), and TTA was determined using the amount of NaOH 1 N needed to achieve a pH value of 8.2 in the samples. The acidity was calculated, taking ascorbic acid as the predominant acid in the strawberries using the mathematical relationship (1).

(1) $$\%\frac{w}{v}\;\mathrm{ascorbic}\;\mathrm{acid}=\frac{N\times V_{1}\times Eq}{V_{2}\times 1000}\times 100$$

where

$N=\mathrm{Standard}\;\mathrm{concentration}\;\mathrm{NaOH}\;\left(\mathrm{normality}\right)$
$V_{1}=\mathrm{Volume}\;\mathrm{NaOH}\;\mathrm{used}$
$V_{2}=\mathrm{Sample}\;\mathrm{volume}$
$Eq=\mathrm{Ascorbic}\;\mathrm{acid}\;\mathrm{equivalent}\;\mathrm{weight}\;\left(0.00277\right).$

#### 3.4.4. Total Soluble Solids (TTS)

The samples of treated and untreated strawberries (control) were blended into juice, and large particulates were separated from the supernatant by decantation. Total soluble solids were determined using a refractometer (Atago ATC-1, Tokyo, Japan) at a temperature of 20 ± 1 °C. Three drops of strawberry juice were taken to obtain the measurements. The refractometer was calibrated to zero with distilled water between each measure [52].

#### 3.4.5. Microbial Stability

At all the sample points, the strawberries were blended into juice. The juice was left to sit for the decantation of the particulates. The supernatant was transferred into a sterilized flask, serially diluted with peptone water 1% (3M, Kamen, Germany), and spread-plated on Yeast and Mold Agar (YMA, Millipore, Milwaukee, WI, USA) for yeast and mold counts, and Plate Count Agar (PCA, Merck, UK) for the total mesophilic aerobic count. The plates were incubated at 25 °C for 24 to 48 h or until colonies were observed [14].

### 3.5. Antioxidant Activity

#### 3.5.1. Total Phenolic Content (TPC) and Ascorbic Acid

Following the manufacturer’s instructions, the TPC and ascorbic acid were determined using an enzymatic autoanalyzer photometer (Y15, Biosystem, Barcelona, Spain).

#### 3.5.2. Flavanol and Anthocyanin Determination

The determination of total flavonoids was conducted following the method described by Zhishen, Mengcheng, and Jianming (1999), with some modifications. Briefly, 1 mL of strawberry ethanolic extract was mixed with 6.4 mL of deionized water, 300 µL of NaNO_2_ at 0.05 g/mL, 300 µL of AlCl_3_ at 0.10 g/mL, and 2 mL of NaOH. The mixture was left to settle for 30 min, and then absorbance was measured at 415 nm in a UV-Vis spectrophotometer (Spectroquant, modelPharo 300, Darmstadt, Germany) [60]. The concentration of total flavonoids was calculated based on a quercetin standard curve, and the results were expressed as mg of quercetin equivalents per g of fresh weight of the sample (mg EQ/g).

Monomeric anthocyanins were determined by a spectrophotometric method reported by [61]. Briefly, 2 mL of the strawberry juice was diluted in 25 mL of a solution composed of 15 mL of 0.2M KCl and 375 mL of 0.2M HCl to achieve a pH of 1. Another 2 mL was diluted up to 25 mL with a buffer solution at pH 4.5 (400 mL of 1M CH_3_CO_2_Na, 240 mL of 1M HCl, and 360 mL of H_2_O). The absorbance of the solutions was measured at 510 nm. The concentration of anthocyanins was determined by:

(2) $$\mathrm{Monomeric}\;\mathrm{anthocyanins}=\frac{\left({Abs}_{pH1}-{Abs}_{pH4.5}\right)*484.82*1000}{\varepsilon }*DF$$

where 484.22 is the molecular weight of Cyanidin-3-glucoside chloride, ε is the molar absorptivity at 510 nm in the pH 1 solution, and DF is the dilution factor.

### 3.6. DPPH Radical Scavenging Assay

One hundred μL of diluted juice was mixed with 1 mL of 2,2-diphenyl-1-picrylhydrazyl (DPPH) (Calbiochem, Merck, Darmstadt, Germany) (100 mg/L). The mixture was incubated in the dark at 37 °C for 45 min. After this time, the samples were centrifuged, and the change in the supernatant color was measured using a spectrophotometer (Spectroquant, modelPharo 300, Darmstadt, Germany) at 517 nm; 100 μL of methanol in 1 mL DPPH was used as the control, and methanol alone was used as the blank. The radical scavenging activity (RSA) was calculated with the formula:

$$\%\;RSA=\left(\mathrm{absorbance}\;\mathrm{of}\;\mathrm{control}-\mathrm{absorbance}\;\mathrm{of}\;\mathrm{sample}\right)*\frac{100}{\mathrm{absorbance}\;\mathrm{of}\;\mathrm{control}}$$

### 3.7. ORAC Antioxidant Activity

The ORAC activity was determined using 2,2′-azobis(2-amidinopropane) dihydrochloride (AAPH) and fluorescein, as described by [62]. Briefly, 45 μL of strawberry juice (diluted in 75 mM phosphate buffer, pH 7.4) was transferred to microplates containing 75 μL of APPH (18 mM) and 200 μL of fluorescein (108 nM). The plates were placed in a Multi-Mode Microplate Reader (Fluoroskan Ascent, Thermo Scientific, Waltham, MA USA) and incubated for 60 min at 37 °C, shaking every 3 min. During incubation, the fluorescence (485 nm Ex/538 nm Em) was monitored every 3 min throughout the experiment. The ORAC activity was expressed as micromoles of Trolox equivalents (TE) per 100 g of dried weight (μmol of TE·100 g/DW).

### 3.8. Sensory Analysis

Twenty-five untrained panelists (average age 25 years old, 13 female and 12 male) were recruited to conduct a 9-point hedonic scale sensory test by scoring the following attributes: texture, color, aroma, sweetness, acidity, and overall acceptance. Fresh decay-free strawberries were washed with sterile water, air-dried, and then dipped in the bioactive coating solution (1% chitosan with 8 mg/mL free extract). After dipping for 1 m, the samples were air-dried and used for evaluation. Strawberries without treatment (non-coated) were used as controls. The samples were presented in white plastic disposable dishes labeled with a 3-digit random code. The panelists were asked to rate each sample on a scale from 1 to 9, where 1 represents “dislike extremely”, and 9 “like extremely”.

### 3.9. Statistical Analysis

All the experiments were carried out in triplicate and two experiment runs. The data were expressed as the mean ± standard deviation. A one-way analysis of variance (ANOVA) was performed to determine significant differences, and for multiple comparisons, the post hoc t-test with Bonferroni correction was used. Statistically significant differences were considered at *p* values < 0.05.

## 4. Conclusions

Our results suggest that coating fresh strawberries with a bioactive coating composed of Arrayan extracts embedded in a chitosan matrix significantly improves the shelf-life of the fruits when stored at 10 °C. After three weeks of storage, the BC prevented significant weight loss, while the strawberries maintained their turgor. The fruits showed little microbial spoilage (without any visible mold formation). The strawberries were able to preserve their antioxidant activity, as reflected by the minor variations in polyphenols, flavonols, anthocyanins, and ascorbic acid concentrations. Finally, the treated strawberries did not change in appearance or flavor, conserving the natural fruits’ sensory characteristics. These results suggest that Arrayan extracts can be used as natural additives to improve the shelf-life of strawberries when embedded in a polymeric chitosan matrix.

## Figures and Tables

**Table 1:** Phenolic composition of the ethanolic Arrayan extracts.

Compound	Formula	PubChem CID	Concentration (mg/L)
Flavonoids			
Quercetin-3-rutinoside	C_27_H_30_O_16_	5280805	718 ± 63.0
Anthocyanins			
Petunidin-3-arabinoside	C_21_H_21_ClO_11_	91810653	121 ± 3.00
Peonidin-3-galactoside	C_22_H_23_ClO_11_	91810512	438 ± 7.00
Peonidin-3-arabinoside	C_21_H_21_ClO_10_	91810651	65.0 ± 2.00
Malvidin-3-arabinoside	C_22_H_23_ClO_11_	91810654	592 ± 5.00

**Table 2:** Antimicrobial activity of free Arrayan extract, chitosan solution, and bioactive coating against common foodborne bacteria and spoilage yeast.

Microorganism	Inhibition Zone Diameter (mm)		
	FE	CH	BC
Foodborne bacteria			
*St. aureus*	21.70 ± 0.03 ^a^	12.30 ± 0.11 ^b^	19.33 ± 0.31 ^c^
*L. innocua*	25.00 ± 0.05 ^a^	13.00 ± 0.11 ^b^	17.17 ± 1.33 ^c^
*E. coli*	22.60 ± 0.04 ^a^	10.30± 0.10 ^b^	17.85 ± 0.21 ^c^
*S. typhymurium*	21.60 ± 0.01 ^a^	13.30 ± 0.10 ^b^	18.32 ± 0.10 ^c^
Yeasts			
*P. kluyverii*	27.10 ± 0.02 ^a^	19.60 ± 0.12 ^b^	19.50 ± 0.03 ^b^
*M. pulcherrima*	28.40 ± 0.04 ^a^	23.50 ± 0.08 ^b^	21.40 ± 0.05 ^b^

Data are presented as mean values ± standard deviations. Different letters within a row represent significant differences (*p* < 0.05). FE: free extract (0.8% *w*/*v*), CH: chitosan solution (1% *w*/*v*), and BC: bioactive coating (1.0% *w*/*v* chitosan + 0.8% *w*/*v* free extract).

**Table 3:** Microbiological stability.

	Treatment	Weeks			
		0	1	2	3
Mesophilic aerobic (log CFU/mL)	Control	2.12 ± 0.01 ^aA^	1.14 ± 0.03 ^aB^	3.39 ± 0.04 ^aC^	4.26 ± 0.02 ^aD^
	FE	1.83 ± 0.04 ^bA^	1.05 ± 0.03 ^aA^	2.23 ± 0.06 ^bB^	3.46 ± 0.04 ^bC^
	CH	2.32 ± 0.02 ^aA^	1.85 ± 0.01 ^aB^	2.52 ± 0.02 ^bA^	3.17 ± 0.01 ^bC^
	BC	1.63 ± 0.06 ^bA^	1.25 ± 0.02 ^aA^	1.37 ± 0.03 ^cA^	1.62 ± 0.01 ^cA^
Yeast and Mold (log CFU/mL)	Control	1.35 ± 0.12 ^aA^	2.11 ± 0.08 ^aB^	3.15 ± 1.10 ^aC^	4.32 ± 0.17 ^aD^
	FE	1.41 ± 0.08 ^aA^	≤0.1 ^bB^	1.21 ± 0.11 ^bA^	3.34 ± 0.18 ^bC^
	CH	1.37 ± 0.11 ^aA^	≤0.1 ^bB^	1.67 ± 0.08 ^bA^	2.78 ± 0.05 ^cC^
	BC	1.32 ± 0.09 ^aA^	≤0.1 ^bB^	1.13 ± 0.02 ^bA^	1.05 ± 0.01 ^dA^

Data are presented as mean values ± standard deviations. Values with different lowercase letters are significantly different (*p* < 0.05) in the same column. Mean values with different capital letters are significantly different (*p* < 0.05) in the same line. FE: free extract (0.8% *w*/*v*); CH: chitosan solution (1% *w*/*v*); BC: bioactive coating (0.8% *w*/*v* FE in 1% *w*/*v* CH solution).

**Table 4:** Antioxidant capacity in strawberries after 21 days of storage at 10 °C.

Treatment	(μmol of TE·100 g/dw) *			
	DPPH		ORAC	
	Initial	Week 3	Initial	Week 3
Control	167.2 ± 1.14 ^aA^	49.50 ± 1.14 ^aB^	75.1 ± 0.12 ^aC^	61.1 ± 0.34 ^aD^
BC	315.4 ± 1.10 ^bA^	264.1 ± 1.24 ^bB^	306.2 ± 2.31 ^bC^	289.1 ± 3.22 ^bC^

Data presented as mean values ± standard deviations. * Micromoles of Trolox equivalents (TE) per 100 g of dried weight. Mean values with different lowercase letters are significantly different (*p* < 0.05) in the same column. Mean values with different capital letters are significantly different (*p* < 0.05) in the same line.

**Table 5:** Concentrations of antioxidant agents in treated strawberries over time.

Treatment	Flavonoids (mg eq/100 g)		Polyphenols (mg EAF/100 g)		Anthocyanins (mg/100 g)		Ascorbic Acid (mg/100 g)	
	Initial	Week 3	Initial	Week 3	Initial	Week 3	Initial	Week 3
Control	1.31 ± 0.12 ^aA^	0.67 ± 0.19 ^aB^	25.2 ± 0.12 ^aA^	7.35 ± 0.09 ^aB^	89.6 ± 0.14 ^bB^	75.2 ± 0.12 ^bA^	97.1 ± 0.12 ^aA^	52.5 ± 0.17 ^aB^
BC	2.27 ± 0.12 ^bA^	1.95 ± 0.16 ^bA^	25.3 ± 0.13 ^aA^	20.6 ± 0.04 ^bB^	101.2 ± 0.21 ^aA^	100.2 ± 0.15 ^aA^	97.2 ± 0.11 ^aA^	80.2 ± 0.15 ^bB^

Data presented as mean values ± standard deviations. Values with different lowercase letters are significantly different (*p* < 0.05) in the same column. Mean values with different capital letters are significantly different (*p* < 0.05) in the same line. Control: without treatment; BC: bioactive coating (0.8% *w*/*v* free extract in 1% *w*/*v* chitosan solution).

## Data Availability

Available at request to the corresponding authors.
